# Use of oral health care services in the United States: unequal, inequitable—a cross-sectional study

**DOI:** 10.1186/s12903-021-01708-8

**Published:** 2021-07-23

**Authors:** Xiangqun Ju, Gloria C. Mejia, Qiang Wu, Huabin Luo, Lisa M. Jamieson

**Affiliations:** 1 Australian Research Centre for Population Oral Health, Adelaide Dental School, The University of Adelaide, Adelaide Health and Medical Sciences Building, Adelaide, 5005 Australia; 2 SA Aboriginal Chronic Disease Consortium, Wardliparingga, South Australian Health and Medical Research Institute (SAHMRI), Adelaide, Australia; 3 Department of Biostatistics, College of Allied Health Sciences, East Carolina University, Greenville, USA; 4 Department of Public Health, Brody School of Medicine, East Carolina University, Greenville, USA

**Keywords:** Inequality, Inequity, Indices of inequality, Concentration curves (CC)

## Abstract

**Background:**

Social determinants drive disparities in dental visiting. Disparities can be measured simply by comparing outcomes between groups (inequality) but can also consider concepts of social justice or fairness (inequity). This study aimed to assess differences in dental visiting in the United States in terms of both social inequality and inequity.

**Methods:**

Data were obtained from a cross-sectional study—the National Health and Nutrition Examination Survey (NHANES) 2015–2016, and participants were US adults aged 30+ years. The outcome of interest, use of oral health care services, was measured in terms of dental visiting in the past 12 months. Disparity was operationalized through education and income. Other characteristics included age, gender, race/ethnicity, main language, country of birth, citizenship and oral health status. To characterize existing inequality in dental service use, we examined bivariate relationships using indices of inequality: the absolute and relative concentration index (ACI and RCI), the slope index of inequality (SII) and relative index of inequality (RII) and through concentration curves (CC). Indirect standardization with a non-linear model was used to measure inequity.

**Results:**

A total of 4745 US adults were included. Bivariate analysis showed a gradient by both education and income in dental visiting, with a higher proportion (> 60%) of those with lower educational attainment /lower income having not visited a dentist. The concentration curves showed pro-higher education and income inequality. All measures of absolute and relative indices were negative, indicating that from lower to higher socioeconomic position (education and income), the prevalence of no dental visiting decreased: ACI and RCI estimates were approximately 8% and 20%, while SII and RII estimates were 50% and 30%. After need-standardization, the group with the highest educational level had nearly 2.5 times- and the highest income had near three times less probability of not having a dental visit in the past 12 months than those with the lowest education and income, respectively.

**Conclusion:**

The findings indicate that use of oral health care is threatened by existing social inequalities and inequities, disproportionately burdening disadvantaged populations. Efforts to reduce both oral health inequalities and inequities must start with action in the social, economic and policy spheres.

## Background

Timely access to oral health care reduces negative impacts of dental diseases [1]. Despite this evidence, disparities persist in accessing and receiving quality oral health care. Broad social (including education, income and wealth, employment the social environment, public safety and so on), structural (including gender, race and ethnicity, immigration status, geography and more) and political factors drive access to services and health outcomes; this is evidenced by international studies on the social determinants of health [2, 3]. Race and ethnicity, income, education, and gender are relevant social determinants from ethical and policy perspectives [4, 5]. Socioeconomic inequalities are commonly found in oral health status, including dental caries [6], periodontal disease [7], oral health-related quality of life [8]; lower income limits access to dental care/service [9, 10] and lower education is associated with lower oral health literacy [11] and poor oral hygiene behaviours [12].

Oral health equity, defined as the fair distribution of oral health determinants, outcomes, and resources within and between segments of the population, regardless of social standing—should drive health system and policy goals [13]. Achieving this goal entails (1) quantifying disparities in a reliable and transparent way, (2) using the evidence-based research findings to inform policy, and (3) turning policy into action and practice (5). The first task—knowledge generation through reliable measurement—defines the scope of science and research [5]. However, measurement requires conceptual clarity in defining disparities (inequality and inequity).

Inequality is framed as health disadvantage in terms of gaps and/or gradients [5]. These are, in turn, measurable and observable quantities that are widely reported through summary measures or indices that quantify the extent and variability of inequalities [14, 15]. Inequity considers how those with the same need are treated in relation to each other and how those with different needs are treated. Inequity cannot be measured directly because it relies on ethical or value judgements on what is believed to be unfair [14, 16]. Thus, equal treatment of medical/dental needs, irrespective of other characteristics such as income, race, education, is defined as horizontal equity and appropriate unequal treatment of unequals is defined as vertical equity [17–20]. The aim of this study is to measure inequality and inequity in dental visiting for adults in the United States. We hypothesize that unequal and inequitable utilisation of oral health care services is associated with existing social differences in education and income among American adults.

## Methods

This is a cross-sectional study, and the sample was adults over the age of 30 years participating in the National Health and Nutrition Examination Survey (NHANES) 2015–16. NHANES is designed to assess the health and nutritional status, including interviews and physical examinations [21], among adults and children in the United States. Variables were selected from the demographic, oral health and acculturation modules. The de-identified data was obtained from open access sources with no requirement for institutional board ethics approval.

The outcome of interest, use of oral health care services, was measured in terms of dental visiting and was derived from the question ‘When did you last visit a dentist?’ The variable originally included 7 categories from less than 6 months to never; in this analysis, we used visiting within the past 12 months and dichotomised the response options (‘Yes’ vs ‘No’).

Disparity variables (for inequality and inequity) were education and income.

Education was based on the highest level of school completed, according to the ‘Education Level-Adults 20+’ classification [21], and categorised as ‘less than high school’, ‘high school’, ‘some college’ and ‘college’.

The income variable was total annual household income (summed across all household members) and categorised as ‘< $20,000’, ‘≥ $20,000 to < $45,000’, ‘≥ $45,000 to < $100,000’ and ‘≥ $100,000’. There were 7.5% missing data for ‘Income’ which could not be assumed to be missing at random, therefore these missing data were not included in the analyses.

Sociodemographic characteristics included gender, race/ethnicity, age, main language spoken at home, country of birth and citizenship.

The original race/ethnicity variable included the following categories: Mexican–American (MA), other Hispanic, non-Hispanic White (NHW), non-Hispanic Black, non-Hispanic Asian (NHA), and another race including multiracial. For the present analysis, MA and other Hispanic were combined into one group and NHA and other or multiracial in another group.

Age was categorised into ‘≥ 30 to < 40 years’, ‘≥ 40 to < 50 years’, ‘≥ 50 to < 60 years’, ‘≥ 60 to < 70 years’ and ‘≥ 70 years’ groups. The target population was adults over the age of 30 because at this age there is a greater likelihood of socio-economic independence and stability (e.g., not dependent on parental income and having completed schooling).

Main language merged two variables: (1) Language at interview which was either Spanish or English and (2) whether an interpreter was required during the interview; it was categorised as ‘English’ or ‘Other’.

Country of birth defined whether the person was born in the US or born in other countries.

Citizenship status indicated whether the person was a US citizen or not.

Need for oral health care was represented by self-rated oral health and impaired oral health.

The NHANES question for self-rated oral health asked participants for an overall rating of the health of their teeth and gums. Self-rated oral health was analysed including the following three categories (1) excellent or very good, (2) good, and (3) fair or poor.

Impaired oral health combined NHANES questions for pain, difficulty at work or school and embarrassment due to problems in teeth, mouth or dentures. The original NHANES questions were related to the frequency of the events (very often, fairly often, occasionally, hardly ever or never). This analysis used a binary variable, with impairment defined as one or more responses of ‘very often’, ‘fairly often’ or ‘occasionally’.

### Data analysis

For all analyses, the outcome was defined as NOT visiting the dentist in the last 12 months.

#### Inequality in dental visits

To characterize existing inequality in the use of oral health care services, we examined bivariate relationships, used indices of inequality and depicted inequalities through concentration curves (CC). The measures of inequality included absolute and relative estimates using the health disparity calculator from SEER—The Surveillance Epidemiology and End Results Program of the US National Cancer Institute [22]. The range difference and ratio were measured between extreme groups in the social gradient. The absolute concentration index (ACI) and relative concentration index (RCI) were used to measure the extent to which the outcome (i.e., no dental visiting in the past 12 months) is concentrated in a particular group. The ACI is calculated by multiplying the RCI by the mean level of the outcome in the population (μ): ACI = μRCI. Thus, if there is no inequality (i.e., if RCI = 0), the ACI is 0 [23]. The slope index of inequality (SII) and relative index of inequality (RII) are regression-based and assume the relationship between ‘no dental visiting in the past 12 months’ and the disparity variables (i.e., education and income) is in a linear manner. The SII represents the absolute effect on ‘no dental visiting in the past 12 months’ of moving from the lowest socio-economic level to the highest. The SII can be calculated by Weighted Least Squares to allow heteroscedasticity of the error terms [24]. The RII is obtained by dividing the SII by the mean level of ‘no dental visiting in the past 12 months’ in the population [23]. The CC provides a graphical view of how dental visiting varied across education and income [13]. The CC plots the cumulative percentage of ‘no dental visits in the last 12 months’ against the cumulative percentage of the population ranked from the lowest/poorest to the highest/richest education and income. If everyone, irrespective of their educational level and income, had the same value of dental visiting, the concentration curve would overlap with the line of equality; that is, it would be a 45-degree line. If the curve is above the line of equality, it means a higher concentration of ‘‘no dental visiting in the past 12 months’ among the lower educational attainment and income groups [13].

#### Inequity in dental visits

Assessing inequity in dental vising requires comparing utilization at various education and income levels for the same level of need; we measured ‘actual’, ‘need-expected (need-predicted)’ and ‘need-standardized’ dental visiting. Actual use was a factual depiction of the extent of inequality in the distribution of dental visiting. Need-expected dental visiting adjusted actual use by self-rated oral health and impaired oral health (i.e., need). The gap between ‘‘actual’ and ‘‘need-expected’ dental visiting reflects different use of oral health care services. Need-standardized use quantified the extent of inequity through the difference between actual and expected use, plus the mean of expected use in the study population [13]. As dental visiting is a binary response, a non-linear model (i.e., Probit regression model) was used with the probability of no dental visiting as the dependent variables to indirectly standardize the dental service utilization [13].

Education and income were strongly associated; therefore, separate models were run for each and the prediction values did not control for each other when presented across levels of education or income. Both models were adjusted for age, sex, race/ethnicity and main language (‘Country of birth’, ‘US Citizen’ and ‘Main language’ were strongly associated, so only the latter was included in the models). ‘Self-rated oral health’ and ‘Impaired oral health’ were strongly associated and therefore, only impaired oral health was used in the models to measure need for oral health care. The assumption in this method is that vertical equity is satisfied—we assume differences in the use of health care among individuals with different needs are appropriate. Any residual inequality after standardization by need for care is interpreted as horizontal inequity.

Weights were used to account for the sampling methodology of the survey; SAS version 9.4 (SAS Institute Inc., Cary, NC, USA) and STATA version 16 (Corporation, College Station, TX, USA) were used for the analyses.

## Results

**Fig. 1:**
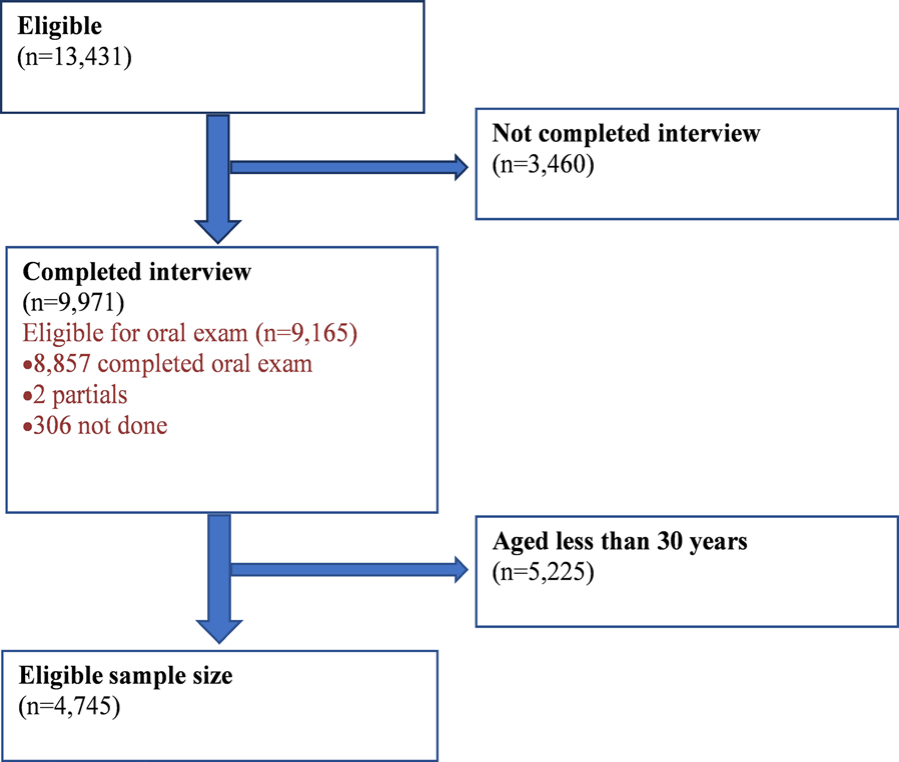
Flow chart on data collection

In the 2015–2016 NHANES, 13,431 people were selected and of those, 9971 completed the interview. This analysis includes data on adults ages 30 and over who responded to the oral health questionnaire; a subsample size of 4745 (see Fig. 1).

Sample characteristics are presented in Table 1. There were slightly more females (52%) and the five age groups were similarly distributed at around 20%. Almost all interviews (91%) were conducted in English and without the use of an interpreter. Approximately two-thirds (66%) of the sample were NHW.

**Table 1:** Sample characteristics, percent with no dental visits (95% CI) (weighted)

	Number	Percent (weighted)	With no dental visits (%)	Difference (95% CI)
Total	4745		39.7	-
*Socio-demographic characteristics*				
Age (years)				
30 to < 40	979	21.6	48.1	Ref
40 to < 50	941	21.4	35.6	− 12.5 (− 16.9, − 8.2)
50 to < 60	924	22.6	42.2	− 5.9 (− 10.4, − 1.4)
60 to < 70	949	18.4	30.4	− 17.7(− 22.0, − 13.5)
70+	952	16.0	40.8	− 7.4 (− 11.8, − 2.9)
Gender				
Female	2458	52.4	36.2	Ref
Male	2287	47.6	43.4	7.2 (4.4–10.0)
Race/ethnicity				
Non-Hispanic White	1574	65.9	36.0	Ref
Non-Hispanic Black	991	10.8	46.5	10.5 (6.6–14.5)
Hispanic	1466	14.0	53.5	17.5 (14.0–20.9)
Other	714	9.3	37.4	1.4 (− 0.0, 0.1)
Main language				
English	3758	90.6	38.0	Ref
Other	987	9.4	55.2	17.2 (13.7–20.6)
Country of birth				
US born	3057	81.0	38.6	Ref
Other countries born	1687	19.0	44.0	5.4 (2.4–8.3)
US citizen				
Yes	3955	90.8	38.0	Ref
No	778	9.2	55.7	17.6 (13.8–21.4)
*Disparity variables*				
Education				
College	1177	33.2	22.0	Ref
Some college	1333	31.3	41.3	19.3 (15.8–22.9)
High school	1004	20.3	50.5	28.4 (24.5–32.3)
Less than high school	1226	15.2	60.3	38.3 (34.7–41.9)
Income				
≥ $100,000	739	27.9	18.8	Ref
$45,000 to < 100,000	1280	32.8	38.6	19.7 (16.1–23.4)
$20,000 to < $45,000	1320	24.2	50.0	31.1 (27.5–34.8)
< $20,000	1050	15.0	60.9	42.1 (38.0–46.2)
*Need*				
Self-rated oral health				
Excellent/very good	1458	39.3	23.3	Ref
Good	1535	31.4	38.2	15.0 (11.7–18.2)
Fair/poor	1746	29.3	63.2	39.9 (36.8–43.0)
Impaired oral health				
Never	3088	69.0	34.6	Ref
Hardly ever	1657	31.0	50.9	16.2 (13.8–21.4)

Approximately 15% were in the less than high school group and 33% in the highest education group. Around 15% had a family income of less than $20,000 and the majority, 33% reported annual incomes between $45,000 and less than $100,000. More than half of the population was split equally between those reporting family incomes ranging from $20,000 to $45,000 and incomes over $100,000.

Around 30% rated their oral health as ‘fair or poor’ and reported problems in at least one of the three measures of impaired oral health. Approximately 40% had not visited a dentist in the prior year (Table 1).

### Inequality

The bivariate analysis indicated clear income gradients in dental visits, with a higher proportion of those on lower income not visiting the dentist. The same held for educational attainment (Table 1). More than 60% of those with less than a high school education reported not visiting a dentist in the prior year whereas 22% of those with at least a college education reported no dental visits during the same time (Table 1).

A higher proportion of males and non-Hispanic Black people reported not visiting a dentist the previous year. The absolute difference between Hispanics and NHW was nearly 18% and the relative difference in the proportion visiting a dentist was 1.5 times higher for NHW than for people who identified as Hispanic. A higher proportion of people 30 to 40 years of age (48%), who spoke another language (55%), were other countries born (44%) and non-US citizens (56%) had not visited a dentist in the last year (Table 1).

The difference in visiting between those with and without impaired oral health was close to 16% or 1.5 times in relative terms; 51% of those with impaired oral health did not have a dental visit in the previous 12 months, whereas 35% without impaired oral health did not visit the dentist. The difference in dental visiting between those who self-rated ‘fair/poor’ and ‘excellent/very good’ was nearly 40% or 2.7 times in relative terms (Table 1).

**Fig. 2:**
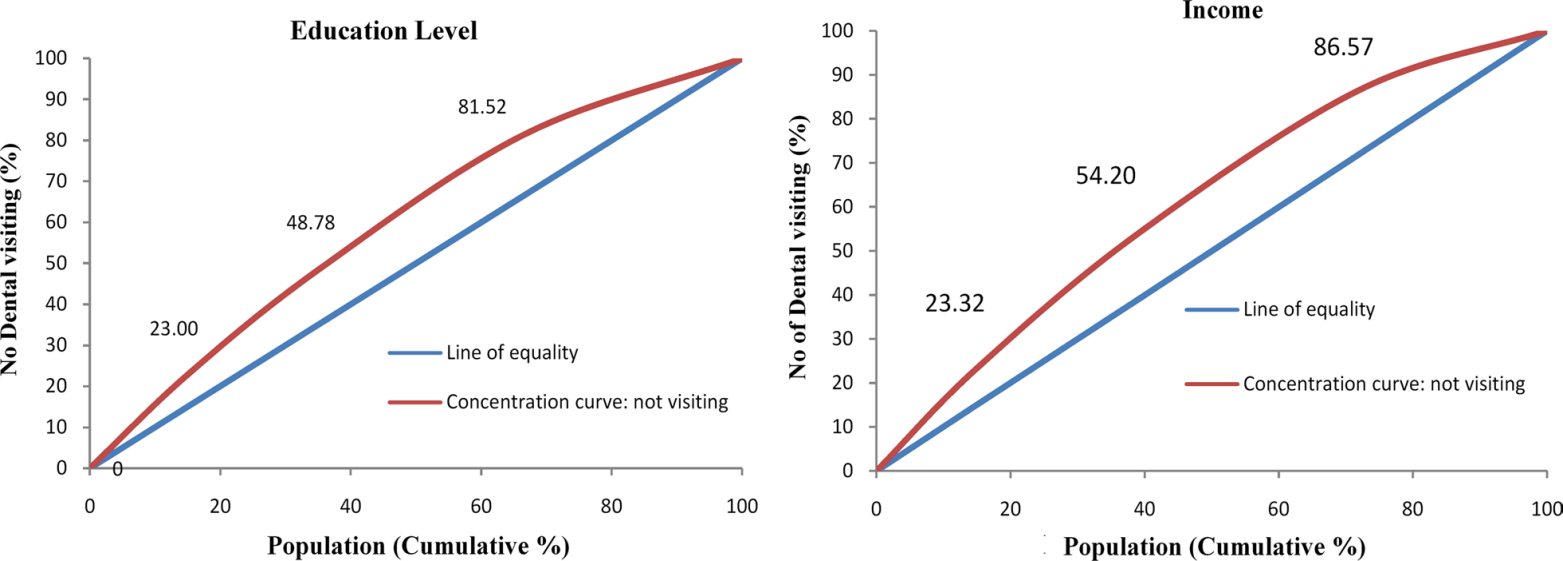
Concentration curves for education and income

The concentration curves (Fig. 2) lie above the 45-degree line of equality, which means there are a pro-higher education and income inequality. In other words, the concentration curves indicated the outcome—not visiting a dentist—takes higher values amongst those with less education and poorer income.

All measures of absolute and relative indices were negative, meaning the prevalence of no dental visiting decreased from lower to higher socioeconomic status. In other words, negative absolute indices indicated pro-higher education/income inequality. SII indicated a difference of around 50 percentage points between a hypothetical individual in the lowest educational level/poorest income and one in the highest educational level/highest income with regard to not visiting a dentist. RII indicates an approximate 30% relative decrease in not visiting when moving from the lowest to the highest educational or income levels. The absolute concentration index (ACI) indicated about 8 percentage points of no dental visiting was concentrated among the groups with lower education or income. The relative concentration index indicates that a redistribution of 20% is required to make the estimated education/income-related inequality equal to zero (Table 2).

**Table 2:** Socioeconomic inequality in dental visits among US adults aged 30 years

Independent variable	Index	Estimate (95% CI)
Education	SII^a^	− 49.76 (− 54.79, − 44.72)
	RII^b^-mean	− 1.26 (− 1.38, 1.13)
	ACI^c^	− 7.63 (− 8.41, − 6.82)
	RCI^d^	− 0.19 (− 0.21, − 0.17)
Income	SII	− 52.46 (− 59.54, − 45.38)
	RII-mean	− 1.34 (− 1.52, − 1.16)
	ACI	− 8.09 (− 9.18, − 7.00)
	RCI	− 0.21 (− 0.24, − 0.18)

^a^The slope index of inequality

^b^The relative index of inequality (mean)

^c^The absolute concentration index

^d^The relative concentration index

### Inequity

Table 3 shows the actual, need-expected and need-standardized distributions for the probability of not having visited a dentist in the previous 12 months by the four levels of education and income**.** Given the outcome is ‘not visiting a dentist’, a negative difference between actual and need-expected means that the probability of actual dental visits is more than the predicted need. For the actual distribution, those with lower education or income have a higher probability of not visiting the dentist. The need-predicted distribution indicates the expectation is that need be equally distributed across all levels of education and income.

**Table 3:** Socioeconomic inequity in dental visits among US adults

Disparity variable	Actual	Need predicted	Difference	Need standardized
Education				
Less than high school	0.6033	0.4504	0.1529	0.5892
High school	0.5046	0.4377	0.0669	0.5032
Some college	0.4130	0.4333	− 0.0203	0.4164
College	0.2204	0.4122	− 0.1918	0.2445
Income				
< $20,000	0.6094	0.4572	0.1522	0.5885
$20,000 to < $45,000	0.4995	0.4410	0.0585	0.4949
$45,000 to < $100,000	0.3856	0.4253	− 0.0397	0.3966
> $100,000	0.1882	0.4104	− 0.2222	0.2142

When we examine the difference between actual non-use (no dental visiting) and predicted non-use based on need, we see that for those with the lowest level of education and income, the probability of not visiting is 15% higher than what would be expected on average given their need. On the other hand, the probability of not visiting a dentist amongst Americans with the highest level of education was 19% lower than what would be expected and for the highest income the probability of not visiting was 22% lower than what would be expected (i.e., people with higher education and income are less likely to not visit the dentist). After need-standardization, the group with the highest educational level and highest income had 2.5 times and nearly 3 times less probability of not having a dental visit in the previous year than those with the lowest educational level and income, respectively. In other words, we could say that if all Americans were to report equal need, those with lower educational levels and income would be less likely to visit the dentist, despite their need, whereas those with higher educational levels and income would visit more than expected based on their reported need.

## Discussion

Our findings support the hypothesis that unequal and inequitable utilisation of oral health services is associated with existing social differences in education and income among American adults. Our study confirms inequalities in the use of oral health care affecting disadvantaged populations, regardless of the outcome measure used. Likewise, regarding inequity, all measures indicate an inverse relationship between the proportion of dental health service use and need: greater use among those with higher education and income, but greater need among those with lower education and poorer income.

Income-related inequity in dental visiting was observed among American adults. Our finding was consistent with other countries, such as in Canada and Brazil [25, 26]. The dental literature is however rich in explaining plausible mechanisms and links:

- Financial barriers were associated with avoiding dental visiting. For instance, without dental insurance, people have delayed dental health care [27], whereas healthcare insurance coverage was positively associated with the likelihood of dental care utilization [28, 29]. However, dental coverage varies with changes in employment, retirement status and poverty [30] – when losing a health benefit, individuals would likely have to pay out of pocket for needed dental care.
- Access barriers affected American adults’ ability to access dental care. Due to budget shortfalls, many US states reduced public health funding for safety net dental clinics [31] and limited dental benefits for poor adults under Medicaid, leading to declining rate of dental visiting for lower income adults [32]. In addition, access barriers also include a shortage of dentists and remoteness [33]. Americans living in rural areas had lower dental utilization and were more likely to report unmet dental needs [27, 34, 35]. Of the 62 million Americans living in rural areas, 43% lacked access to regular oral health care [36].

Education-based disparities in dental visiting was also observed in American adults, which had similar findings: higher educational levels are reported to be related to more dental visiting [37]. This was consistent across the globe [38], and possible explanations included:

- There is an interaction between education and economic position, which determines an individual’s financial, such as affecting private insurance, result in a pro-rich disparity [24, 38]
- There is a closely interlink between education and oral health literacy. Lower education level association with lacking oral health-related knowledge, leading to underutilization of oral health care services [38].

Although race/ethnicity factor was adjusted in our study, it is worth further in-depth study and discussion. Racial and ethnic disparities in the utilization of dental care remain high in the United States with Hispanic and Non-Hispanic Black populations being almost 3 times less likely to have a dental visit during the previous year than the Non-Hispanic White population [27, 39]. Another finding indicated that when accounting for residential segregation African American people had greater use of dental services than white people living in the same environment [40]. The latter highlights the importance of structural factors and further supports the role of social determinants in health. Widespread public health insurance coverage or implementation of pro-equity policies could result in the decline of dental health care inequalities and inequity [12, 41].

In addition to the large and representative sample size, the strengths of the study were to move beyond the measurement of differences (inequalities) by accounting for need to quantify inequities in dental service use, thereby offering policy relevant information.

There are limitations to our study. (1) The study is a secondary data analysis limiting the availability in number and types of variables. (2) Cross-sectional data require careful examination of the temporality of each variable and the timing of the relationship between variables. For example, impaired oral health (measure of need) and dental visiting were both measured within the past 12 months, which is conceptually acceptable, yet, we intentionally did not include clinical data in the analysis, because the clinical information would have occurred after the one-year period of dental visiting, hence not meeting the temporality criteria. (3) Missing values from ‘income’ (7.5%) might lead to biased estimates, however, data analysis by using weighted data with a large simple size would reduce random error.

In order achieve the goal of equity in the use of oral health care serves, future health disparity research also should identify key factors associated with education/income and lack of dental service use to provide the necessary evidence-base to inform policy change.

## Conclusion

The findings indicate that the use of oral health care is threatened by existing social inequalities and inequities, disproportionately burdening disadvantaged populations. Considering the need for services contributes to a better understanding of oral health disparities. It provides tangible evidence of the social differences in dental visiting by indicating that people who need dental care are less likely to visit the dentist and receive the care they need, this is marked by socioeconomic standing. Such approach advances health disparities research by moving beyond the simple measurement of existing gaps in the use of dental services, which provides no information based on the need for services. Efforts to reduce oral health inequalities and most importantly, oral health inequities must start with action in the social and political spheres.

## Data Availability

The datasets generated during and/or analyzed during the current study are available in the: https://wwwn.cdc.gov/nchs/nhanes/continuousnhanes/default.aspx?BeginYear=2017.

## Abbreviations

ARC: The absolute concentration index
CC: The concentration curves
MA: Mexican-American
NHA: Non-Hispanic Asian
NHW: Non-Hispanic White
NHANES: The National Health and Nutrition Examination Survey
OHRQoL: Oral health-related quality of life
RCI: The relative concentration index
RII: The relative index
SII: The slope index
US: The United States
